# Xanthine Oxidase Inhibitory Potential, Antioxidant and Antibacterial Activities of *Cordyceps militaris* (L.) Link Fruiting Body

**DOI:** 10.3390/medicines6010020

**Published:** 2019-01-29

**Authors:** Tran Ngoc Quy, Tran Dang Xuan

**Affiliations:** Graduate school for International Development and Cooperation, Hiroshima University, Hiroshima 739-8529, Japan; tnquy@ctu.edu.vn

**Keywords:** *Cordyceps militaris*, xanthine oxidase, antioxidant, antibacterial, cordycepin, GC-MS

## Abstract

**Background:***Cordyceps militaris* is a medicinal mushroom and has been extensively used as a folk medicine in East Asia. In this study, the separation of constituents involved in xanthine oxidase (XO) inhibitory, antioxidant and antibacterial properties of *C. militaris* was conducted. **Methods:** The aqueous residue of this fungus was extracted by methanol and then subsequently fractionated by hexane, chloroform, ethyl acetate and water. The ethyl acetate extract possessed the highest XO inhibitory and antioxidant activities was separated to different fractions by column chromatography. Each fraction was then subjected to anti-hyperuricemia, antioxidant and antibacterial assays. **Results:** The results showed that the CM8 fraction exhibited the strongest XO inhibitory activity (the lowest IC_50_: 62.82 μg/mL), followed by the CM10 (IC_50_: 68.04 μg/mL) and the CM7 (IC_50_: 86.78 μg/mL). The level of XO inhibition was proportional to antioxidant activity. In antibacterial assay, the CM9 and CM11 fractions showed effective antibacterial activity (MIC values: 15–25 mg/mL and 10–25 mg/mL, respectively). Results from gas chromatography-mass spectrometry (GC-MS) analyses indicated that cordycepin was the major constituent in the CM8 and CM10 fractions. **Conclusions:** This study revealed that *C. militaris* was beneficial for treatment hyperuricemia although in vivo trials on compounds purified from this medicinal fungus are needed.

## 1. Introduction

Species in the genus *Cordyceps* are considered as valuable traditional medicines and other medical applications worldwide, especially in East Asia countries [1,2]. Among them, *Cordyceps militaris* (L.) Link is an ancient medicinal tonic and the most of *C. militaris* nowadays is produced by various modern culture techniques [3]. *C. militaris* exhibited a wide spectrum of clinical health benefits including antifatigue and antistress [4]; anti-inflammatory [5]; antiviral [6]; antifungal and anticancer [7]; HIV-1 protease inhibitory [8]; antioxidant [9]; anti-microbial [10]; inhibition high-fat diet metabolic disorders [11]; immunomodulatory [12]; anti-tumor and anti-metastatic activities [13]. 

Furthermore, the hot water extract of *C. militaris* has been reported to contain various important bioactive compounds such as cordycepin, adenosine, polysaccharides, fatty acids, mannitol, amino acids, trace elements, ash, fiber and other chemical compositions [7,9,10,14,15,16,17]. Many researchers noted that cordycepin (3′-deoxyadenosine) is an important and active metabolite [2,18]. The fermented broth of *C. militaris* obtains clinical effects such as the prevention of alcohol-induced hepatotoxicity [19], inhibitory effects on proliferation and apoptotic cell death for human brain cancer cells [20], inhibitory effects on LPS-induced acute lung injury [21], anti-hyperglycemia [22], anti-tumor and anti-metastatic activities [17]. Adenosine, another bioactive chemical of *C. militaris*, has a number of pharmacological functions such as cardio-protective and therapeutic agents for chronic heart failure, a homeostatic modulator in the central nervous system [16], antioxidant and HIV-1 protease inhibitory [8]. *C. militaris* also exhibited antifungal [23,24], cytotoxic activity [25], antibacterial, anti-tumor agents [13] and plasma glucose reduction [26]. However, the xanthine inhibitory activity of this fungus has not been comprehensively examined.

Nowadays, hyperuricemia, a pre-disposing factor of gout, has been recognized as a lifestyle syndrome that affects the adult population in the developed as well as developing countries [27]. Gout is induced by overproduction or under-excretion of uric acid. It is caused by a high dietary intake of foods containing high amounts of nucleic acids, such as some types of seafood, meats (especially organ meats) and yeasts [28]. Xanthine oxidase (XO) is considered as a cause of hyperuricemia. The acute hyperuricemia can lead to the development of gout, hypertension, diabetes, chronic heart failure, atherosclerosis and hyperlipidemia [29]. Until now, only allopurinol and febuxostat have been clinically approved as XO inhibitors to treat hyperuricemia and gout. However, they also result in many undesirable effects such as hypersensitivity syndrome, hepatitis nephropathy, eosinophilia, vasculitis, fever, and skin rash [30,31].

The discovery of compounds possessing XO inhibitory is necessary to avoid such adverse effects of allopurinol and febuxostat. Yong et al. [29] found that hot water extract of *C. militaris* exhibited significant anti-hyperuricemic action but active components for this activity were not determined. Additionally, the investigation on antibacterial performance of aqueous extracts of *C. militaris* has been proceeded but bioactive compounds from the methanolic extract have not been elaborated [32,33,34,35]. Infectious diseases caused by bacteria are still the major reason of illness and death in developing countries [36]. Gastroenteritis and urinary tract infection were predominated by bacteria such as *Escherichia coli*, *Staphylococcus aureus*, *Proteus mirabilis*, and *Bacillus subtilis* [37,38]. Many plant extracts have been found as nutritionally safe and easily degradable source of antibacterial agents against human pathogens [39]. Hence, this study was conducted to investigate the xanthine oxidase inhibitory and determine the correlation to the antioxidant and antibacterial properties of the folk medicine *C. militaris*. The analyses of bioactive constituents from this medicinal mushroom were also conducted.

## 2. Materials and Methods 

### 2.1. Chemicals

Methanol, hexane, chloroform, ethyl acetate and ethanol were purchased from Junsei Chemical Co., Ltd., Tokyo, Japan. Potassium phosphate monobasic and dibasic, xanthine, xanthine oxidase, allopurinol, and hydrochloric acid were obtained from Sigma-Aldrich Corp., St. Louis, MO, USA. Reagents including 1,1-diphenyl-2-picrylhydrazyl (DPPH), sodium acetate, acetic acid, 2,2′-azinobis (3-ethylbenzothiazoline-6-sulfonic acid) (ABTS), potassium peroxodisulfate, and dibutyl hydroxytoluene (BHT) were supplied by Kanto Chemical Co. Inc., Tokyo, Japan. Four bacteria including *Staphylococcus aureus, Escherichia coli, Bacillus subtilis*, and *Proteus mirabilis* were provided by Sigma-Aldrich Corp., St. Louis, MO USA. All chemicals used were of analytical grade.

### 2.2. Plant Materials and Samples Preparation

The dried and sterilized fruiting bodies of *C. militaris* were provided by Truc Anh Company, Bac Lieu city, Vietnam. Fruiting body at green house of Truc Anh Company in the South of Vietnam were harvested and dried by freeze-drying machine (Mactech MSL1000, 15 °C) and packaged on April 18th, 2017. The sample was transferred to the Laboratory of Plant Physiology and Biochemistry, Graduate School for International Development and Cooperation (IDEC), Hiroshima University, Higashi-Hiroshima, Japan for further analysis. 

### 2.3. Preparation of Plant Extract 

The whole fruiting body of *C. militaris* was soaked in water for 12 h at room temperature and dried in a convection oven (MOV-212F (U), Sanyo, Japan) at 50 °C for 2 d before pulverized into powder using a grinding machine. The powder (1.0 kg) was immersed in 15 L methanol (MeOH) for two weeks at room temperature. After that, the filtrate from powder-methanol dispersion was concentrated under vacuum at 45 °C using a rotary evaporator (SB-350-EYELA, Tokyo Rikakikai Co., Ltd., Tokyo, Japan) to produce 126.14 g of crude extract. The crude extract was suspended in distilled water (500 mL) and successively fractionated with hexane, chloroform (CHCl_3_) and ethyl acetate (EtOAc) to produce 10.24, 19.25, 50.21, and 20.17 g extracts, respectively. The extract with the highest xanthine oxidase inhibitory and antioxidant activities was used for further separation by column chromatography. 

### 2.4. Fractionation of Ethyl Acetate Fraction 

The EtOAc extract (16.28 g) possessed the highest xanthine oxidase inhibitory and antioxidant on a preliminary test was subjected to a normal-phase of column chromatography (40 mm diameter × 600 mm height, Climbing G2, Mixell, Tokyo, Japan) filled with silica gel (size Ǻ 60, 200–400 mesh particle size, Sigma-Aldrich, Tokyo, Japan). This process yielded 14 fractions by increasing the polarity by MeOH with CHCl_3_ of the following eluents: CM1 in CHCl_3_, CM2 in CHCl_3_:MeOH (9.9:0.1), CM3 in CHCl_3_:MeOH (9.8:0.2), CM4 in CHCl_3_:MeOH (9.6:0.4), CM5 in CHCl_3_:MeOH (9.4:0.6), CM6 in CHCl_3_:MeOH (9.2:0.8), CM7 in CHCl_3_:MeOH (9:1), CM8 in CHCl_3_:MeOH (8.8:1.2), CM9 in CHCl_3_:MeOH (8.6:1.4), CM10 in CHCl_3_:MeOH (8.4:1.6), CM11 in CHCl_3_:MeOH (8:2), CM12 in CHCl_3_:MeOH (7:3), CM13 in CHCl_3_:MeOH (1:1), and CM14 in CHCl_3_:MeOH (4:6). 

### 2.5. Xanthine Oxidase (XO) Inhibitory Activity

The XO inhibitory activity was examined spectrophotometrically in aerobic conditions as described previously [40] with some adjustments. The assay mixture consisted of 50 µL of tests solution (6.25–100.00 µg/mL), 30 µL of 70 mM phosphate buffer (pH = 7.5) and 30 µL of enzyme solution (0.01 units/mL in 70 mM phosphate buffer, pH = 7.5), which were prepared immediately before use. After pre-incubation at 25 °C for 15 min, reaction was initiated by addition of 60 µL of substrate solution (150 µM xanthine in buffer). After that, the assay mixture was incubated at 25 °C for 30 min. The reaction was stopped by adding 25 µL of 1 N hydrochloric acid (HCl) and the absorbance was measured at 290 nm by using a microplate reader (Multiskan^TM^ Microplate Spectrophotometer, Thermo Fisher Scientific, Osaka, Japan). A blank was prepared in similar way but the enzyme solution was accumulated to the assay mixture after the solution of 1 N HCl added. One unit of XO was defined as the amount of enzyme that required to produce 1 µmol of uric acid per min at 25 °C.

The XO inhibitory activity was calculated by this formula (1):

(1) $$\%\;\mathrm{Inhibition}=\left\{\frac{\left(A-B\right)-\left(C-D\right)}{\left(A-B\right)}\right\}\times 100$$

where A was the activity of the enzyme without test extracts or fractions, B was the control of A without test extracts or fractions and enzyme. C and D were the activities of the test solutions with and without XO. The values of IC_50_ were calculated from the means of the spectrophotometric data of the test trials repeated 5 times. The test solutions were dissolved in DMSO (dimethyl sulfoxide) followed by dilution with buffer. The final concentration of DMSO was less than 0.25%. Allopurinol at 6.25, 12.5, 25, 50, 100 µg/mL dilutions were used as a positive control.

### 2.6. Antibacterial Activity

The evaluation of antibacterial activity was based on a method described previously [41]. All bacterial strains were cultured in a Luria-Bertani (LB) broth for 24 h at 37 °C. The four bacterial strains employed in this experiment included *Staphylococcus aureus*, *Escherichia coli*, *Bacillus subtilis* and *Proteus mirabilis*. The final population was standardized to be 1.29 × 10^6^ CFU/mL (*S. aureus*), 1.45 × 10^6^ CFU/mL (*E. coli*), 1.63 × 10^6^ CFU/mL (*B. subtilis*) and 2.87 × 10^6^ CFU/mL (*P. mirabilis*). An amount of 0.1 mL of the bacteria suspension was spread over the surface of the solid LB agar medium in Petri dish (9 cm in diameter). After that, filter paper discs (6 mm diameter) loaded with 20 µL of each extract or fraction sample (with a concentration 40 mg/mL in DMSO) were placed on the surface of the LB agar plates. The Petri dishes were incubated at 37 °C for 24 h and then the inhibition zone was measured. Ampicillin and streptomycin were used as the positive controls. The concentrations of the fractions included 1.25, 1.5, 2.5, 5, 10, 20, 25, 30, and 40 mg/mL). The lowest concentration that inhibited the visible bacterial growth was evaluated as minimal inhibitory concentration (MIC). Ampicillin and streptomycin (1.25, 0.625, 0.313, 0.156, 0.078, 0.039, 0.0195, 0.0097, 0.0048, 0.0024, 0.0012, and 0.0006 mg/mL) were used as positive controls. Subsequently, DMSO was used as a negative control.

### 2.7. Antioxidant Activity

#### 2.7.1. DPPH Radical Scavenging Activity

The antioxidant activity of the extracts and achieved fractions were determined by using 2,2-Diphenyl1-picrylhydrazyl (DPPH) free radical scavenging method as described previously [42] with some adjustments. Briefly, an amount of 100 μL samples was mixed with 50 μL of 0.5 mM DPPH and 100 μL of 0.1 M acetate buffer (pH 5.5). After mixing, the mixtures were maintained in the dark at room temperature for 30 min. The reduction of the DPPH radical was measured at 517 nm using a microplate reader. BHT standard solutions (0.001–0.05 mg/mL) were used as positive controls (2).

DPPH radical scavenging activity (%) = [{A_control_ − (A_sample_ − A_blank sample_)}/A_control_] × 100 (2)

where A_control_ was the absorbance of DPPH solution without samples. A_sample_ was the absorbance of sample with DPPH solution and A_blank sample_ was the absorbance of sample without DPPH solution. Lower absorbance showed higher DPPH radical scavenging activity. The IC_50_ (inhibitory concentration) value was determined as the concentration required to decrease the initial DPPH radical concentration by 50%. Therefore, the lower IC_50_ value indicated higher DPPH radical scavenging activity. 

#### 2.7.2. ABTS Radical Scavenging Activity

The ABTS radical cation decolorization assay was carried out as an improved ABTS method mentioned noted previously [43] with some modifications. Briefly, the ABTS radical solution was prepared by mixing 7 mM ABTS [2,20-azinobis (3-ethylbenzothiazoline-6-sulfonic acid)] and 2.45 mM potassium persulfate in water. After that, this solution was incubated in the dark at room temperature for 16 h and then diluted with methanol to obtain an absorbance of 0.70 ± 0.05 at 734 nm. An aliquot of 120 μL of the ABTS solution was mixed with 24 μL of samples and the mixture was incubated in the dark at room temperature for 30 min. The absorbance of reaction was recorded at 734 nm using a microplate reader. BHT standard (0.01–0.25 mg/mL) was used as a reference. The percentage inhibition was calculated according to the formula (3):

ABTS radical scavenging activity (%) = [{A_control_ − (A_sample_ − A_blank sample_)}/A_control_] × 100 (3)

The A_control_ was the absorbance of ABTS radical solution without samples. A_sample_ was the absorbance of ABTS radical solution with samples and A_blank sample_ was the absorbance of sample without ABTS radical solution. A lower absorbance therefore indicated higher ABTS radical scavenging activity. The IC_50_ (inhibitory concentration) value was calculated as the concentration needed to scavenge 50% of ABTS. As a result, lower IC_50_ value showed higher antioxidant activity. 

### 2.8. Identification of Chemical Constituents by Gas Chromatography-Mass Spectrometry (GC-MS) 

A volume of 1 µL aliquot of each *C. militaris* fraction was injected into a GC-MS system (JMS-T100 GCV, JEOL Ltd., Tokyo, Japan). The column employed in this experiment was DB-5MS column (length 30 m, internal diameter 0.25 mm, thickness 0.25 µm) (Agilent Technologies, J & W Scientific Products, Folsom, CA, USA). The system uses helium as a carrier gas and the split ratio was 5.0/1.0. The temperature program was set up in the GC oven as follows: the initial temperature at 50 °C without hold time, the programmed rate by 10 °C/min up to a final temperature of 300 °C with 20 min for hold time. The injector and detector temperatures were set at 300 °C and 320 °C, respectively. The mass range scanned from 29–800 amu. The peak data set was collected by using the JEOL’s GC-MS Mass Center System version 2.65a (JEOL Ltd., Tokyo, Japan) and by comparing detected peaks with National Institute of Standards and Technology (NIST) MS library [44].

### 2.9. Statistical Analysis

The data were statistically analyzed by one-way ANOVA using the Minitab 16.0 software (Minitab Inc., State College, PA, USA). The significant difference among means were determined by using Fisher’s test with the confidence level of 95% (*p* < 0.05). All experiments were carried out in triplicate and expressed as means ± standard deviation (SD). 

## 3. Results

### 3.1. Xanthine Oxidase Inhibitory Activity of C. militaris Fractions

Xanthine oxidase inhibition, which resulted in a decreased of uric acid production, was measured spectrophotometrically at 290 nm. The ethyl acetate extract (EtOAc extract) showed an xanthine oxidase inhibition by 31.66% at 100 μg/mL concentration, whereas other extracts exhibited negligible inhibitions (Table 1). 

All of 14 fractions from the EtOAc extract were assessed for their xanthine oxidase inhibitory ability. Of them, eight fractions showed the presence of XO inhibition activity (Table 2). Furthermore, the percentage of XO inhibition of CM8 (52.58%), CM7 (52.72%), CM10 (56.56%), and CM6 (61.70%) fractions were found to be more active than other fractions. The XO inhibition were described by IC_50_ value and the lower IC_50_ indicated the higher XO inhibition activity. Therefore, the CM8 fraction possessed the most potential XO inhibition (IC_50_, 62.82 μg/mL), followed by CM10 (IC_50_, 68.04 μg/mL), CM7 (IC_50_, 86.78 μg/mL), and CM6 (IC_50_, 87.73 μg/mL) fractions. Other fractions exhibited trivial inhibitory activities which were not considerable enough to calculate IC_50_ values.

### 3.2. Antioxidant Activities of C. militaris Fractions 

**Figure 1:**
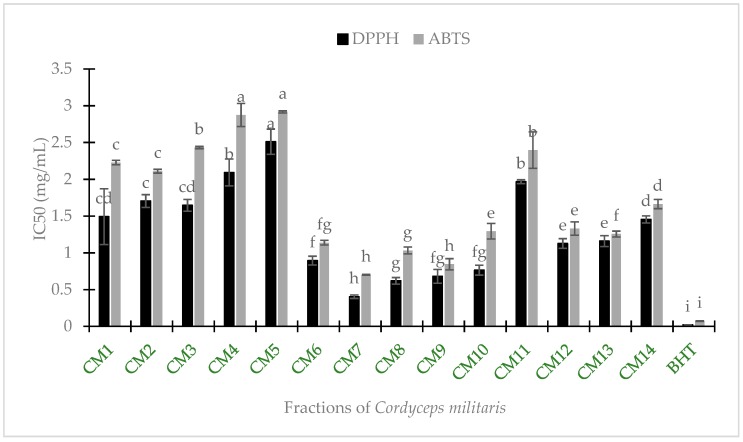
DPPH and ABTS radical scavenging activities of fractions from *C. militaris* and standard antioxidant butylated hydroxytoluene (BHT). Column with similar letters are not significantly different (*p* < 0.05).

The antioxidant activities of *C. militaris* were evaluated using DPPH and ABTS tests, compared with the standard BHT in Figure 1. The antioxidant properties were described by IC_50_ value and the lower IC_50_ indicated the higher radical scavenging activity. Fourteen fractions obtained from *C. militaris* showed various levels of DPPH and ABTS scavenging capacity (Table 1), of which the fraction CM7 presented the strongest antioxidant activity in both DPPH and ABTS assays. Meanwhile, the antioxidant activity of CM5 was the lowest performance in Figure 1.

In ABTS scavenging activity, the fractions CM7 and CM9 exposed the highest effective activity (IC_50_, 0.702 mg/mL and 0.845 mg/mL, respectively), followed by the CM8 (IC_50_, 1.032 mg/mL) and CM6 (IC_50_, 1.138 mg/mL). In the DPPH assay, the CM8 was also potential but it was statistically similar to that of the CM9 and CM10. Overall, it was found the CM6, CM7, CM8, and CM9 possessed greater antioxidant capacities than other fractions.

### 3.3. Antibacterial Activities of C. militaris Extracts 

The antibacterial activity of *C. militaris* was conducted on two Gram-positive (*B. subtilis* and *S. aureus*) and two Gram-negative (*E. coli* and *P. mirabilis*). Table 3 showed that levels of antibacterial activities versus four bacteria were varied among fractions. Both CM9 and CM11 were the most potential candidates to inhibit the growth of most tested bacteria (MIC, 15–25 mg/mL and 10–25 mg/mL). All fractions showed a lower inhibition than that of streptomycin and ampicillin. Ampicillin and streptomycin provided MIC values of 0.0097–0.039 and 0.078–0.156 mg/mL (Table 3). 

### 3.4. GC-MS of Analysis of C. militaris 

Gas chromatographic-mass spectrometry (GC-MS) is a very powerful and reliable analytical technique for identifying the presence of constituents in complex mixtures [45]. The major active components of the principal 14 fractions were detected and identified by GC-MS (Supplementary Materials Figures S1–S14) and summarized in Table 4. Principal constituents from *C. militaris* included cordycepin (3′-deoxyadenosine), hexadecenoic acid and pentadecanal. (Table 4; Supplementary Materials Figures S1–S14). 

Cordycepin, appeared as the main compound that was detected in fractions of CM8, CM9 and CM10, while pentadecanal was found in most of fractions (CM3-CM10). Additionally, fatty acids (hexadecanoic acid, methyl hexadecanoate and methyl 2-oxohexadecanoate) were distributed in the CM1, CM2, CM6, CM7, CM11, CM12, CM13 and CM14 fractions. (Table 4; Supplementary Materials Figures S1–S14).

## 4. Discussion

It was reported that the significant increase of gout and hyperuricemia principally caused by the changes in unusual habits of diet and exercise regimen [46]. The food with high content of nucleic acids such as meat and seafood raised the risk of gout disease. Hyperuricemia is a biochemical abnormality or metabolic disorder that results in development of gout and related oxidative stress-related diseases such as cancer, cardiovascular disease and a variety of other disorders [47]. Therefore, the lowering serum uric acid concentration within normal range is important and can be achieved by blocking the biosynthesis of uric acid [27]. Xanthine oxidase (XO) is a form of xanthine oxidoreductase, which has been discovered for decades. Natural XO inhibitors from plants are used in traditional herbal medicines for the treatment of gout or diseases associated with symptoms such as arthritis and inflammation [28]. From this fact, screening of XO inhibitory activity from medicinal plants might be an effective way to find new potential candidates for these major disease treatments. In this study, the xanthine oxidase inhibitory, antioxidant and antibacterial activities of *C. militaris* were determined. It was found that *C. militaris* obtained potent xanthine oxidase inhibitory, antioxidant and antibacterial properties and possessed rich phytochemicals which were characterized by column chromatography and GC-MS analyses (Table 1, Table 2, Table 3 and Table 4). 

Several previous studies showed that the majority of natural compounds that possessed XO inhibition belonged to lanostanoids [48], flavonoids [31], and phenolics [49]. From GC-MS results, cordycepin appeared as the major bioactive constituents in CM8, CM9, and CM10 fractions separated by column chromatography. Thus it was suggested that this compound may be responsible for the XO inhibition, although the purification of cordycepin as well as other bioactive components and examined for their XO inhibition is apparently required. Earlier researches showed that cordycepin obtained remarkable anti-hyperuricemic action in an in vivo model [50]. Thus, this research highlighted that cordycepin found *C. militaris* played a crucial role in inhibition of XO by an in vitro model. Oxidative stress results in human disease development or an abnormal immune response [9]. Furthermore, it was reported that free radicals caused oxidative damage to biomolecules and are responsible for progression of several diseases such as aging, cancer, inflammatory, diabetes, metabolic disorders, atherosclerosis and cardiovascular diseases [51]. Therefore, xanthine oxidase acted as a biological source of oxygen-derived free radical that led to cell and tissue damage [48]. Obviously, the XO inhibitory activity of *C. militaris* was attributed to their survival strategy to the oxidative stress. For example, several studies showed that polysaccharides from aqueous extracts of *C. militaris* possessed antioxidant properties [33,34,35] but there was little polysaccharide quantity found in methanolic extracts [25]. Furthermore, the in vitro antioxidant activity was reported to be correlated to cordycepin [21,52] and fatty acids [53]. The considerable amounts of cordycepin and fatty acids observed in CM7, CM8, CM9 and CM10 fractions by this study noticed that these compounds obtained in *C. militaris* might be responsible for significant antioxidant performance (Table 1; Figure 1) as found in previous reports [23,54]. 

The urinary tract infection and gastroenteritis have become a more serious problem today because of multidrug resistance to *E. coli*, *S. aureus*, *P. mirabilis* and *B. subtilis* infection [37,38]. In recent years, it was documented that methanolic extract of *C. militaris* had potential antibacterial activity [23,25]. To date, thousands of phytochemicals derived from plant extracts with various mechanisms of action have been identified as antibacterial compounds [55]. In this study, cordycepin appeared as the key component antibacterial activity, especially in *E. coli* and *B. subtilis* although further in vitro trial was needed. This study highlighted that *C. militaris* obtained potential substances which may be beneficial for the treatments of gout and bacterial infection. Several previous studies also indicated that fatty acids and the derivative methyl esters exhibited antibacterial activities [53,56]. The fatty acids with a chain length of more than 10 carbon atoms induced lysis of bacterial protoplasts. This mechanism could further distress the expression of bacterial virulence which played an important role in establishing infection [57]. Therefore, the presence of n-hexadecanoic acid (CM12, CM11, CM2, and CM1 fractions), hexadecanoic acid, 2-hydroxy-1-(hydroxymethyl) ethyl ester (CM11), hexadecanoic acid, 2-oxo-, methyl ester (CM9, CM7), hexadecanoic acid, methyl ester and 9,12-octadecadienoic acid methyl ester (Table 4) suggested that these constituents characterized by this study may be responsible for potent antibacterial activity of this medicinal fungus as reported by many previous reports [58,59]. This study has successfully separated fractions from *C. militaris* active on XO inhibitory, antioxidant and antibacterial activities separated by column chromatography and identified potent constituents by GC-MS analysis. However, the minimum bacteria concentration (MBC) should also be measured to achieve more efficacies on antibacterial activity. It was proposed that there were some compounds other than cordycepin and fatty acids in *C. militaris* can also be potential for pharmaceutical properties and needed further analyses. 

## 5. Conclusions

This is the first study revealed that the medicinal fungus *C. militaris* possessed strong xanthine oxidase inhibition which may be potential for hyperuricemia treatment, although further in vivo trial is required. By employing separative techniques of column chromatography and GC-MS analyses, cordycepin, fatty acids and their derivatives appeared as the major compounds that may be responsible for antioxidant, antibacterial and anti-hyperuricemia activities as observed by this research. Findings of this study highlighted that *C. militaris* is potential to develop foods and drinks potential for treatment of hyperuricemia. Investigation of bioactive constituents purified from *C. militaris* on potent medicinal and pharmaceutical properties of this ancient fungus should be further elaborated.

## Figures and Tables

**Table 1:** Xanthine oxidase inhibitory and antioxidant activities of *C. militaris*.

Extracts	% XO Inhibition at 100 µg/mL	Antioxidant Activities	
		DPPH (IC_50_ mg/mL)	ABTS (IC_50_ mg/mL)
Hexane (H)	-	3.07 ± 0.04 ^a^	4.45 ± 0.06 ^a^
Chloroform (C)	-	1.65 ± 0.15 ^b^	2.52 ± 0.19 ^b^
Ethyl acetate (E)	31.66 ± 2.86	0.60 ± 0.03 ^d^	1.03 ± 0.02 ^d^
Aqueous residue (W)	-	1.35 ± 0.07 ^c^	1.65 ± 0.07 ^c^

Data presented means ± standard deviation (SD). Values in a column with similar letters are not significantly different by Fisher’s test (*p* < 0.05). -: not detected.

**Table 2:** Xanthine oxidase inhibitory activity of EtOAc fractions isolated from *C. militaris*.

Fractions	% XO Inhibition at 100 µg/mL	IC_50_ Value (µg/mL)
CM1	-	-
CM2	-	-
CM3	-	-
CM4	21.88 ± 0.78 ^f^	-
CM5	39.57 ± 0.56 ^d^	-
CM6	61.70 ± 0.64 ^b^	87.73 ± 0.81 ^a^
CM7	52.72 ± 0.74 ^c^	86.78 ± 1.20 ^a^
CM8	52.58 ± 1.55 ^c^	62.82 ± 4.48 ^b^
CM9	31.12 ± 3.71 ^e^	-
CM10	56.56 ± 2.95 ^c^	68.04 ± 5.85 ^b^
CM11	-	-
CM12	11.92 ± 1.79 ^g^	-
CM13	-	-
CM14	-	-
Allopurinol	90.20 ± 6.19 ^a^	4.85 ± 2.19 ^c^

Data presented means ± standard deviations (SD). Values in a column with similar letters are not significantly different (*p* < 0.05); -: not detected.

**Table 3:** Antibacterial activity in term of MIC values of EtOAc fractions isolated from *C. militaris*.

Fractions	Minimum Inhibitory Concentration (mg/mL)			
	*B. subtilis*	*S. auereus*	*E. coli*	*P. mirabilis*
CM1	25	25	30	25
CM2	30	20	25	30
CM3	30	-	30	30
CM4	-	-	-	-
CM5	30	30	30	-
CM6	25	20	-	-
CM7	25	30	25	30
CM8	-	30	20	-
CM9	15	25	25	20
CM10	-	30	30	-
CM11	10	25	15	25
CM12	15	20	20	30
CM13	-	20	-	-
CM14	-	25	-	-
DMSO	-	-	-	-
Ampicillin	0.0195	0.039	0.0097	0.0195
Streptomycin	0.156	0.078	0.156	0.156

-: no inhibition.

**Table 4:** Principal compounds identified from different fractions of *C. militaris*.

No.	Major Constituents	Retention Times (min)	Peak Area (%)	Fractions
1	1) Methyl hexadecanoate	16.72	5.95	CM1
	2) Hexadecanoic acid	17.09	17.08	
	3) (9*Z*,12*E*)-Octadeca-9,12-dienoic acid	18.73	29.54	
2	1) Methyl hexadecanoate	16.72	2.23	CM2
	2) Hexadecanoic acid	17.11	20.64	
	3) (9*Z*,12*E*)-Octadeca-9,12-dienoic acid	18.76	32.16	
	4) (9*R*,10*R*,13*R*,17*R*)-17-[(*E*,2*R*,5*R*)-5,6-Dimethylhept-3-en-2-yl]-10,13-dimethyl-1,2,9,11,12,15,16,17-octahydrocyclopenta [a] phenanthren-3-one	29.12	6.25	
3	1) Pentadecanal	14.56	16.02	CM3
	2) Methyl 2-oxohexadecanoate	17.41	3.04	
	3) Octadecanal	22.11	34.91	
	4) Dodecanamide	25.55	2.73	
4	1) Pentadecanal	14.56	10.38	CM4
	2) Methyl 2-oxohexadecanoate	17.40	3.20	
	3) Octadecanal	22.11	30.13	
5	1) Pentadecanal	14.56	7.11	CM5
	2) Hexadecanal	15.65	1.30	
	3) Methyl 2-oxohexadecanoate	17.41	3.22	
	4) Octadecanal	22.11	25.85	
6	1) (1*R*,2*R*,3*S*,4*R*)-3-Deuterio-6,8-dioxabicyclo [3.2.1] octane-2,3,4-triol	11.82	5.65	CM6
	2) Pentadecanal	14.56	53.80	
	3) Hexadecanoic acid	17.07	1.33	
	4) Methyl 2-hydroxyhexadecanoate	20.30	1.25	
	5) Henicosan-1-ol	26.33	1.99	
7	1) (1*R*,2*R*,3*S*,4R)-3-Deuterio-6,8-dioxabicyclo [3.2.1] octane-2,3,4-triol	11.76	1.92	CM7
	2) Pentadecanal	14.52	21.35	
	3) Hexadecanoic acid	17.03	1.75	
	4) Methyl 2-oxohexadecanoate	17.37	1.26	
	5) *N*-(2-Hydroxyethyl) octanamide	18.77	2.73	
8	1) (1*R*,2*R*,3*S*,4*R*)-3-Deuterio-6,8-dioxabicyclo [3.2.1] octane-2,3,4-triol	11.76	0.54	CM8
	2) Pentadecanal	14.52	19.79	
	3) 3′-Deoxyadenosine	21.98	55.38	
9	1) Pentadecanal	14.55	19.90	CM9
	2) Methyl 2-oxohexadecanoate	17.40	0.77	
	3) 3′-Deoxyadenosine	21.97	58.04	
10	1) Tetradecanal	13.39	0.83	CM10
	2) Pentadecanal	14.56	45.00	
	3) 3′-Deoxyadenosine	21.95	18.61	
11	1) 2-hydroxybutanedioic acid	6.18	1.89	CM11
	2) Hexadecanoic acid	17.03	1.90	
	3) (11*E*,13*Z*)-Octadeca-1,11,13-triene	18.67	0.72	
	4) 1,3-Dihydroxypropan-2-yl hexadecanoate	21.89	3.79	
12	1) Hexadecanoic acid	17.03	1.41	CM12
	2) (11*E*,13*Z*)-Octadeca-1,11,13-triene	17.68	1.27	
	3) (1*R*)-1-Hexadecyl-2,3-dihydro-1*H*-indene	21.74	2.62	
13	1) Hexadecanoic acid	17.02	4.18	CM13
	2) (11*E*,13*Z*)-Octadeca-1,11,13-triene	18.67	15.71	
14	1) *N*,*N*-Dimethyl-1-undecanamine	12.06	2.02	CM14
	2) Hexadecanoic acid	17.03	9.95	
	3) (11*E*,13*Z*)-Octadeca-1,11,13-triene	18.76	25.50	

## Supplementary Materials

The following are available online at http://www.mdpi.com/2305-6320/6/1/20/s1, Figure S1: GC-MS spectrum of fraction F1, Figure S2: GC-MS spectrum of fraction F2, Figure S3: GC-MS spectrum of fraction F3–6, Figure S4: GC-MS spectrum of fraction F7–12, Figure S5: GC-MS spectrum of fraction F13–16, Figure S6: GC-MS spectrum of fraction F17–24, Figure S7: GC-MS spectrum of fraction F25–30, Figure S8: GC-MS spectrum of fraction F31–35, Figure S9: GC-MS spectrum of fraction F36–42, Figure S10: GC-MS spectrum of fraction F43–47, Figure S11: GC-MS spectrum of fraction F48–52, Figure S12: GC-MS spectrum of fraction F53–58, Figure S13: GC-MS spectrum of fraction F59–62, Figure S14: GC-MS spectrum of fraction F63–70.
