# Supplementary material for: Xanthine Oxidase Inhibitory Potential, Antioxidant and Antibacterial Activities of Cordyceps militaris (L.) Link Fruiting Body
Source: Medicines (Basel). 2019 Jan 29;6(1):20. doi: 10.3390/medicines6010020 (PMC6473835; doi:10.3390/medicines6010020)
Supplement: Supplementary file 1 [file medicines-06-00020-s001.pdf]

Supplementary Materials: Xanthine Oxidase Inhibitory Potential, Antioxidant and Antibacterial Activities of *Cordyceps Militaris* (L.) Link Fruiting Body

Tran Ngoc Quy and Tran Dang Xuan

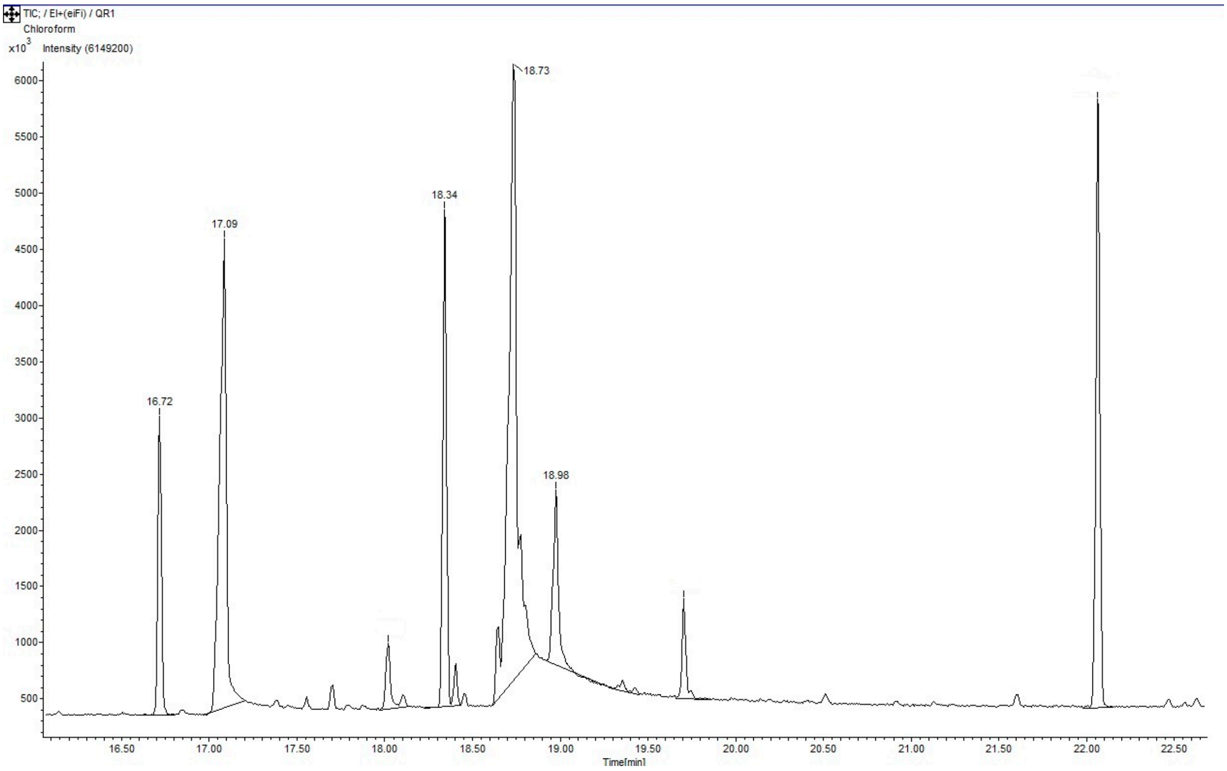

Figure S1. GC-MS spectrum of fraction F1.

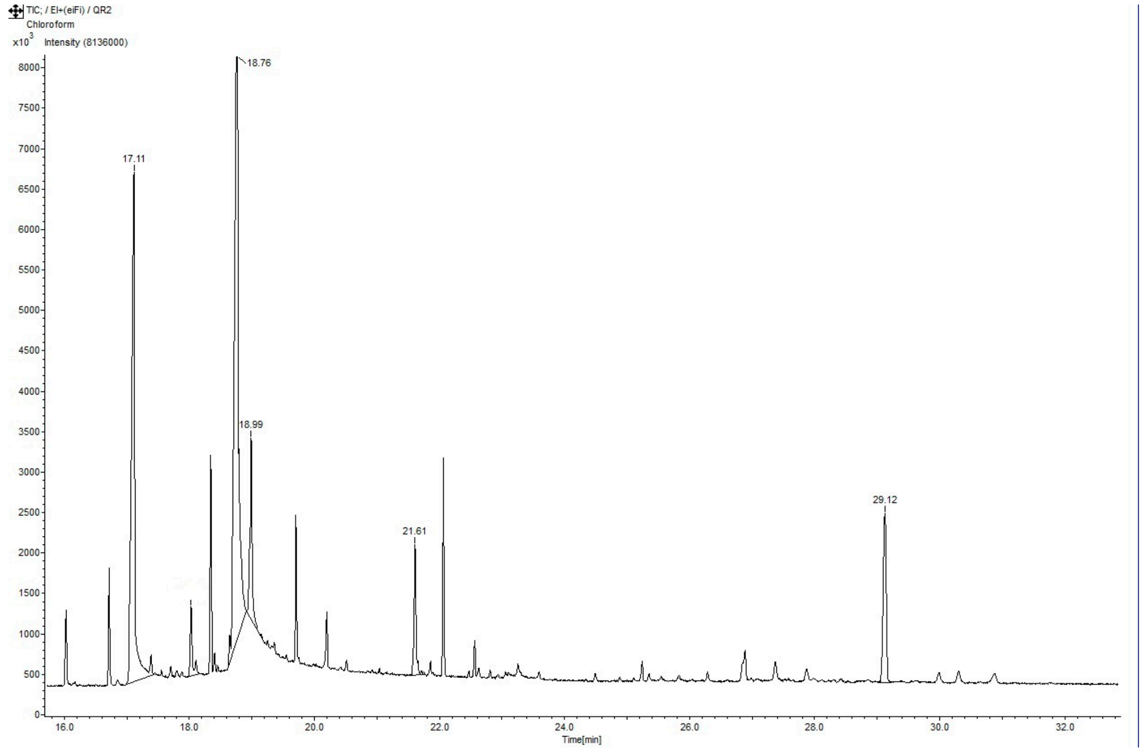

Figure S2. GC-MS spectrum of fraction F2.

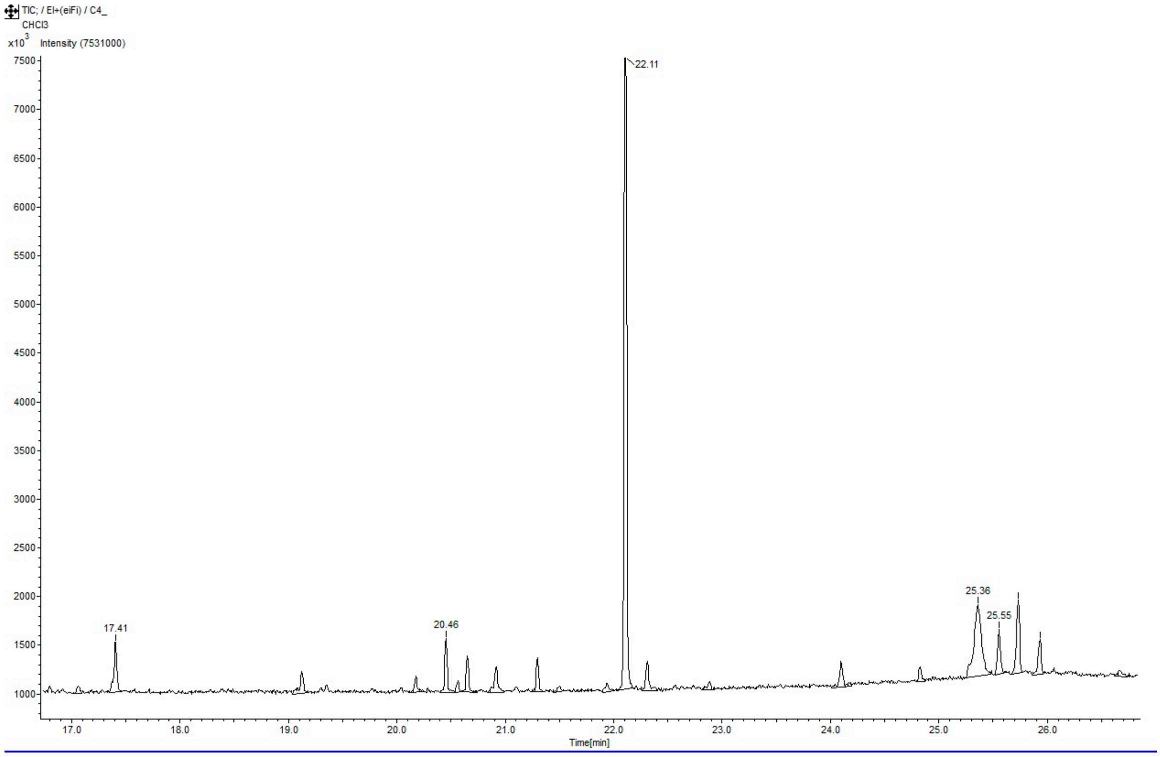

Figure S3. GC-MS spectrum of fraction F3–6.

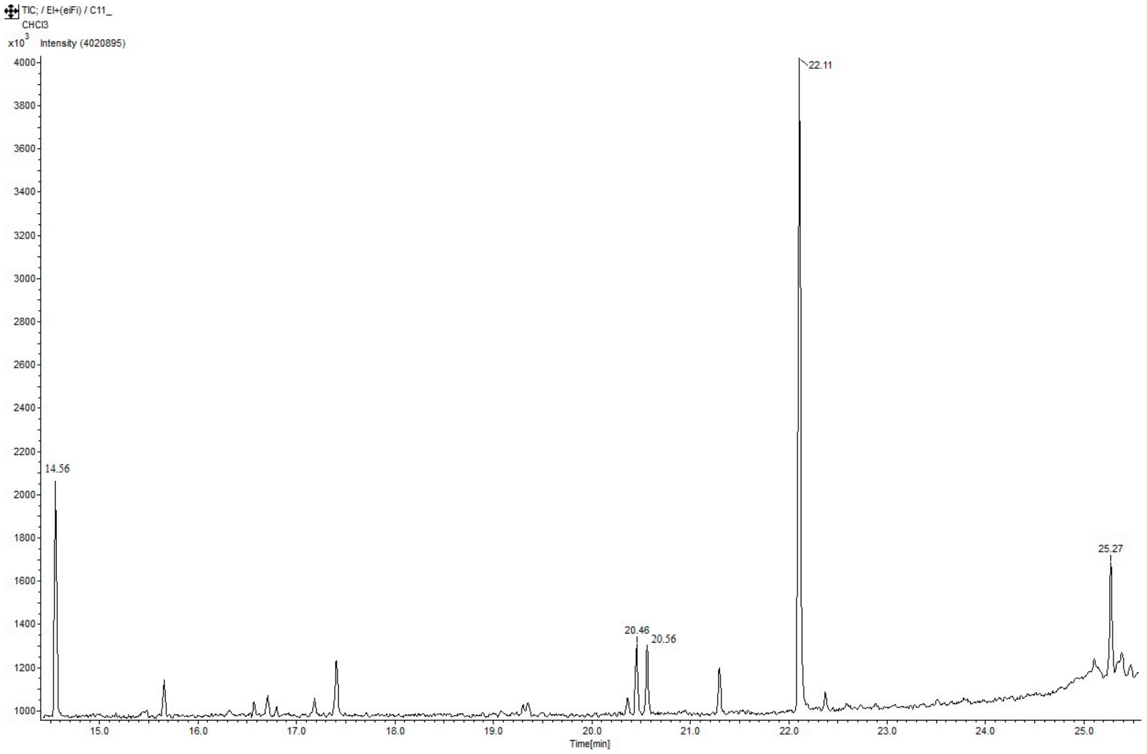

Figure S4. GC-MS spectrum of fraction F7–12.

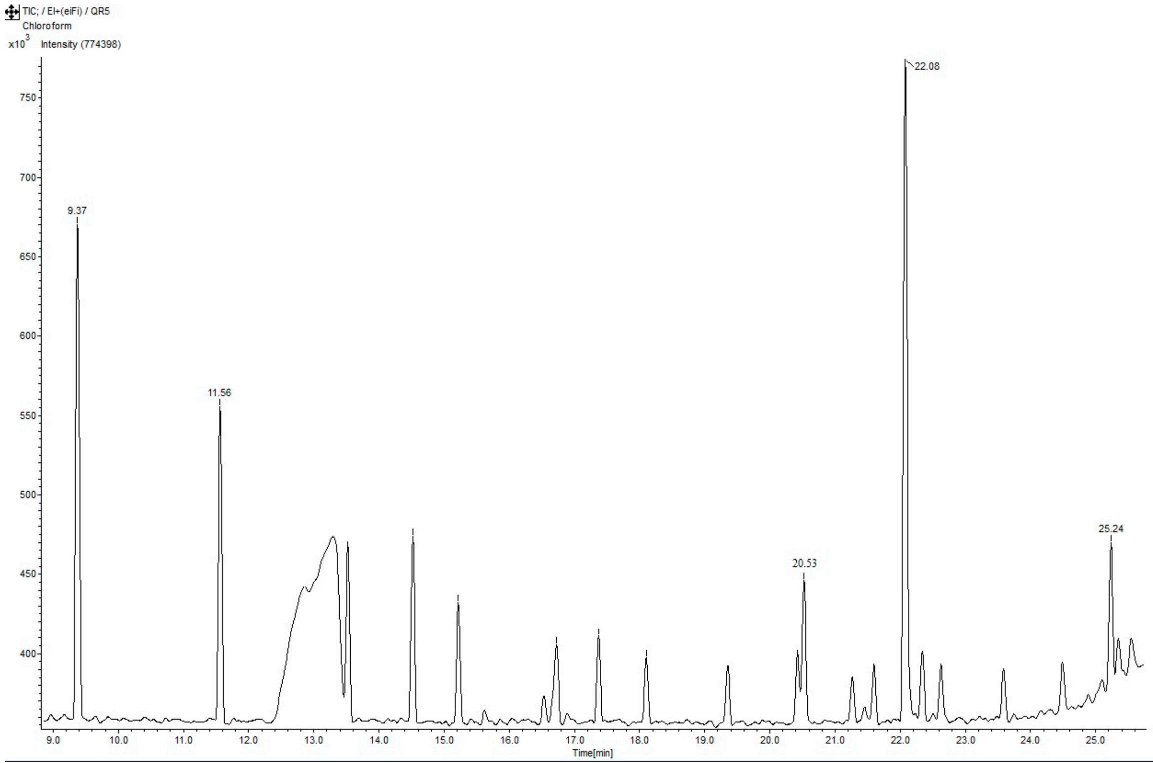

Figure S5. GC-MS spectrum of fraction F13–16.

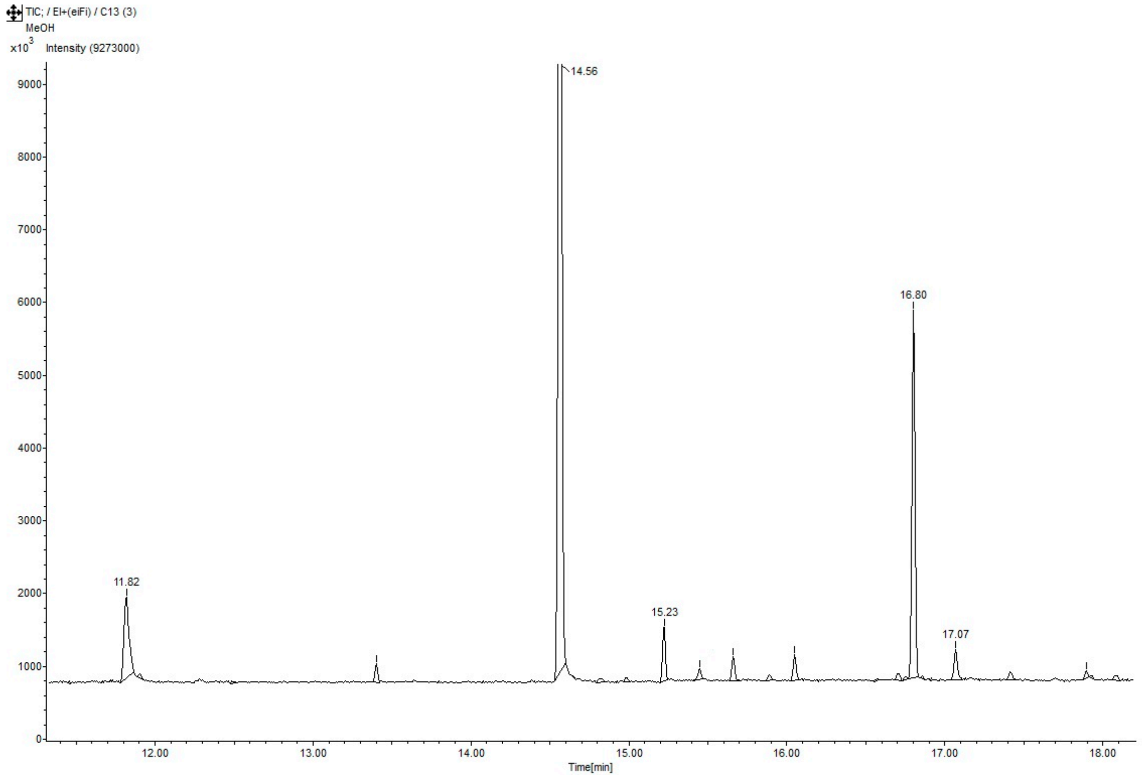

Figure S6. GC-MS spectrum of fraction F17–24.

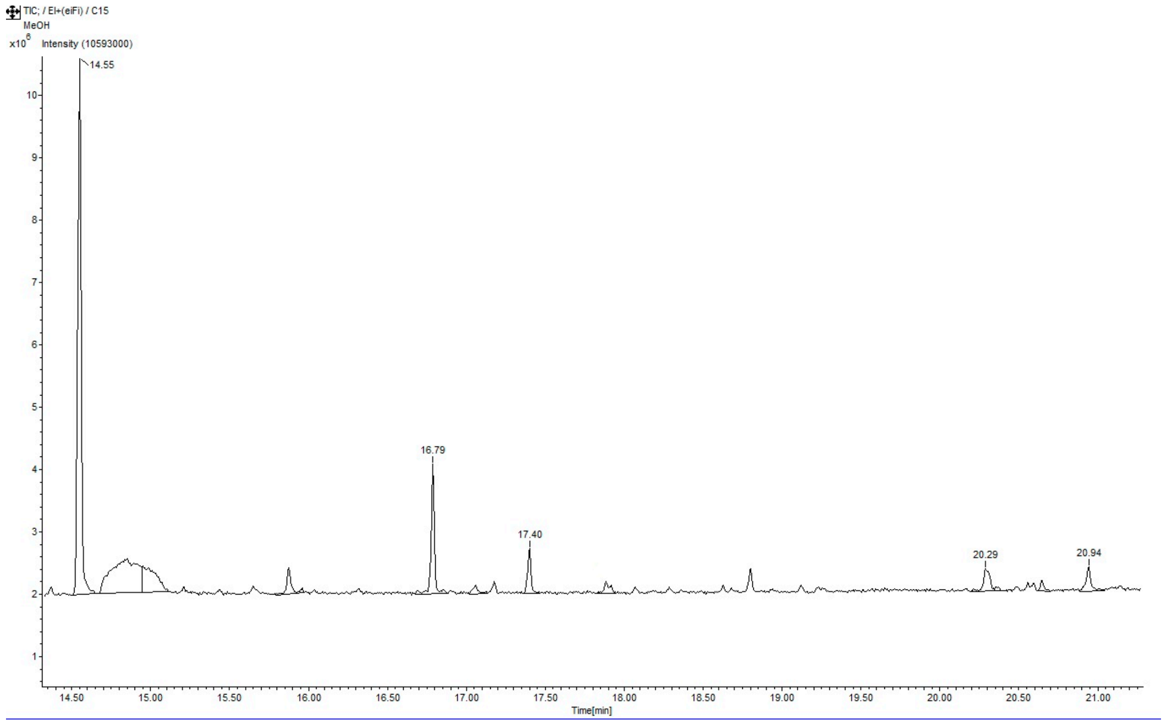

Figure S7. GC-MS spectrum of fraction F25–30.

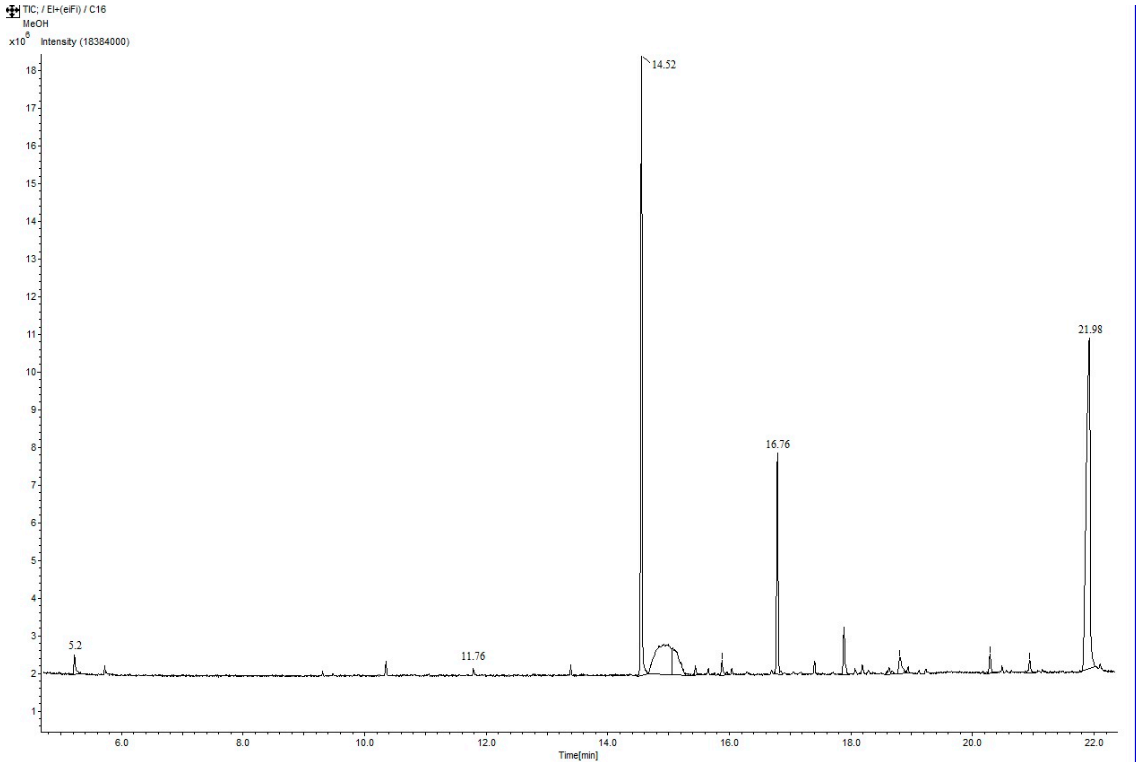

Figure S8. GC-MS spectrum of fraction F31–35.

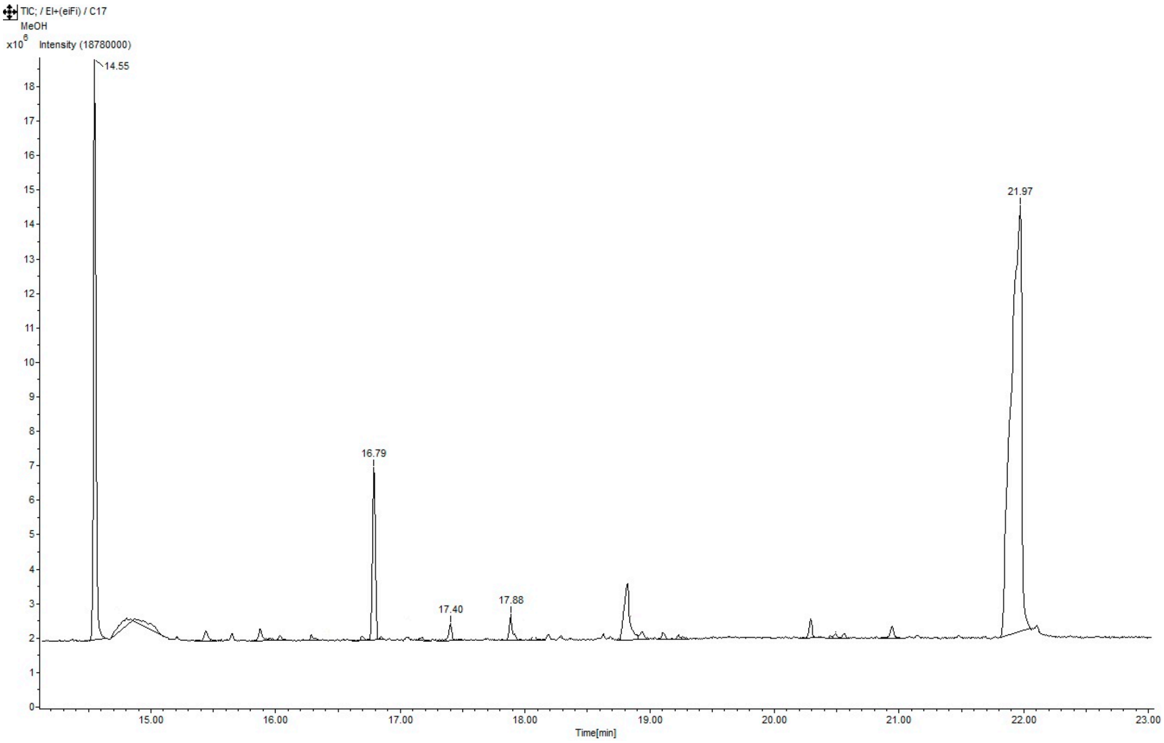

Figure S9. GC-MS spectrum of fraction F36–42.

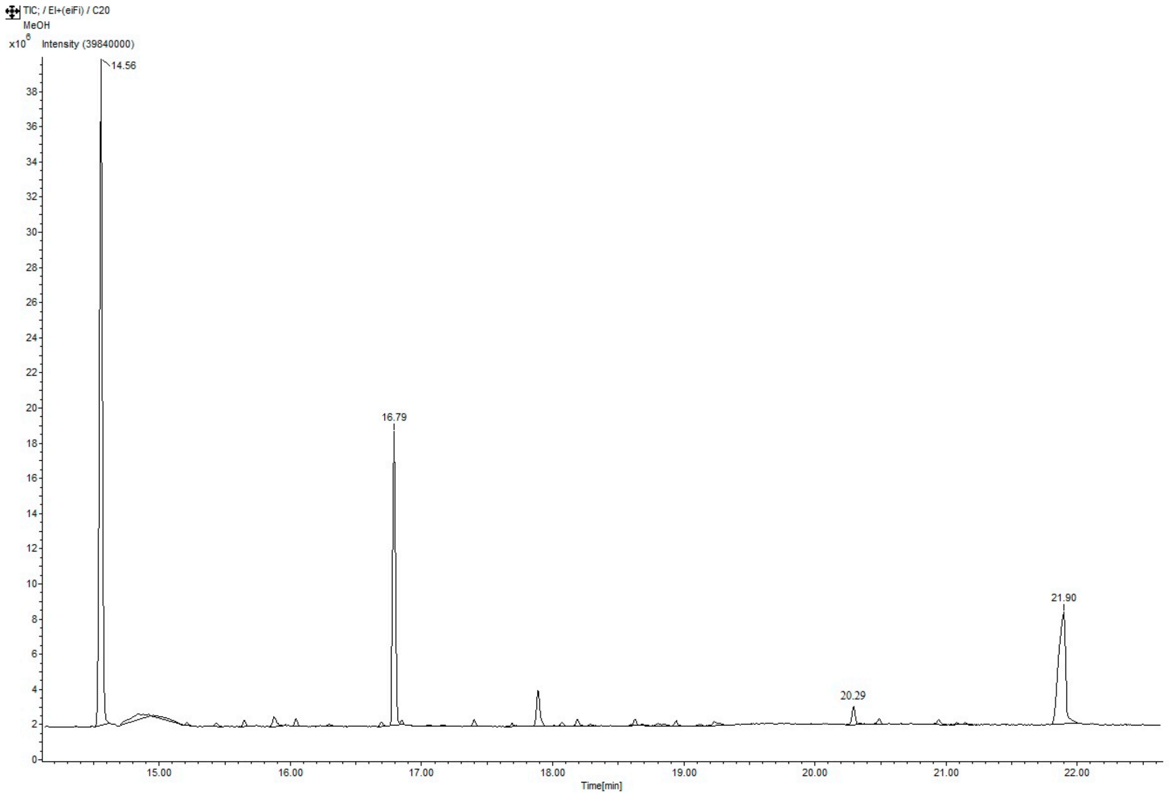

Figure S10. GC-MS spectrum of fraction F43–47.

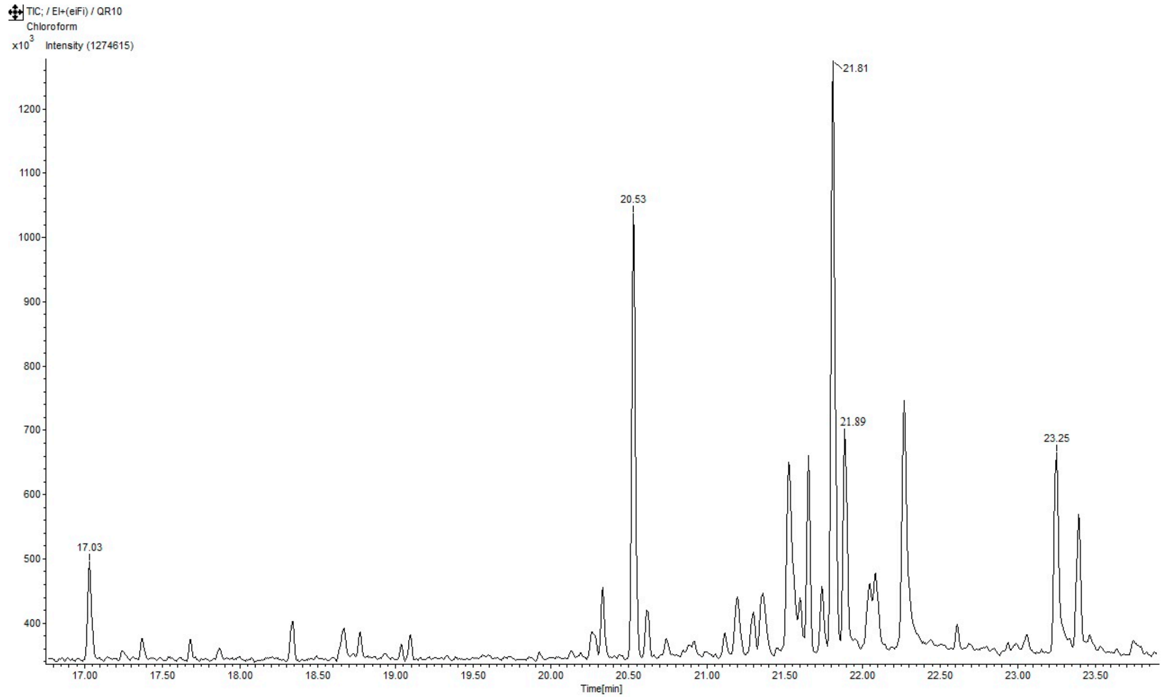

Figure S11. GC-MS spectrum of fraction F48–52.

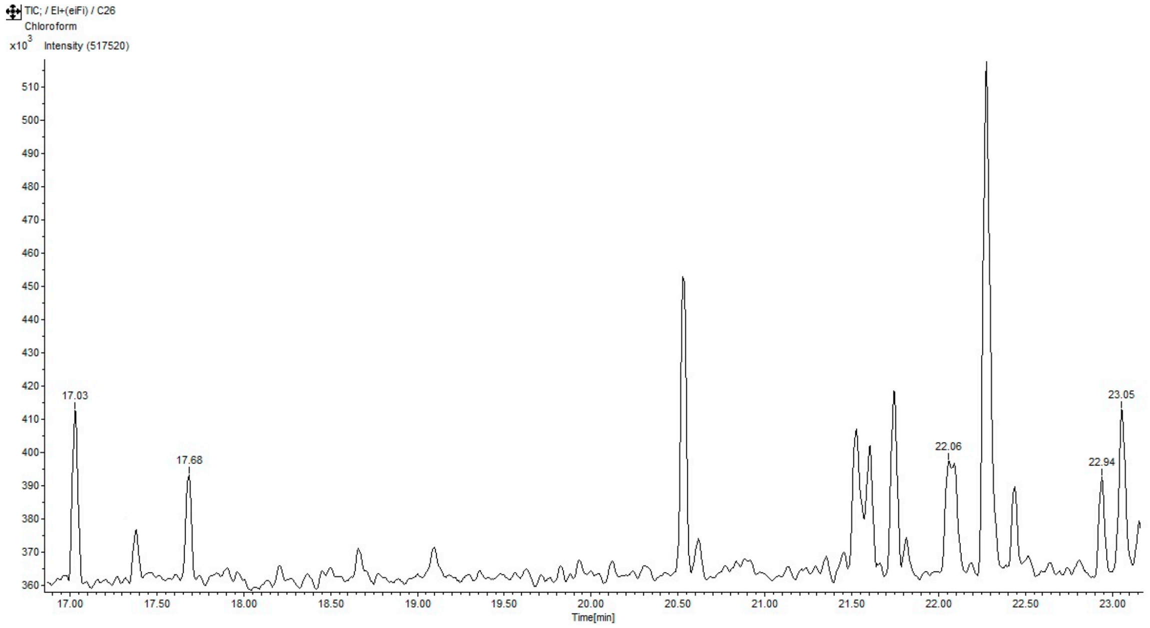

Figure S12. GC-MS spectrum of fraction F53–58.

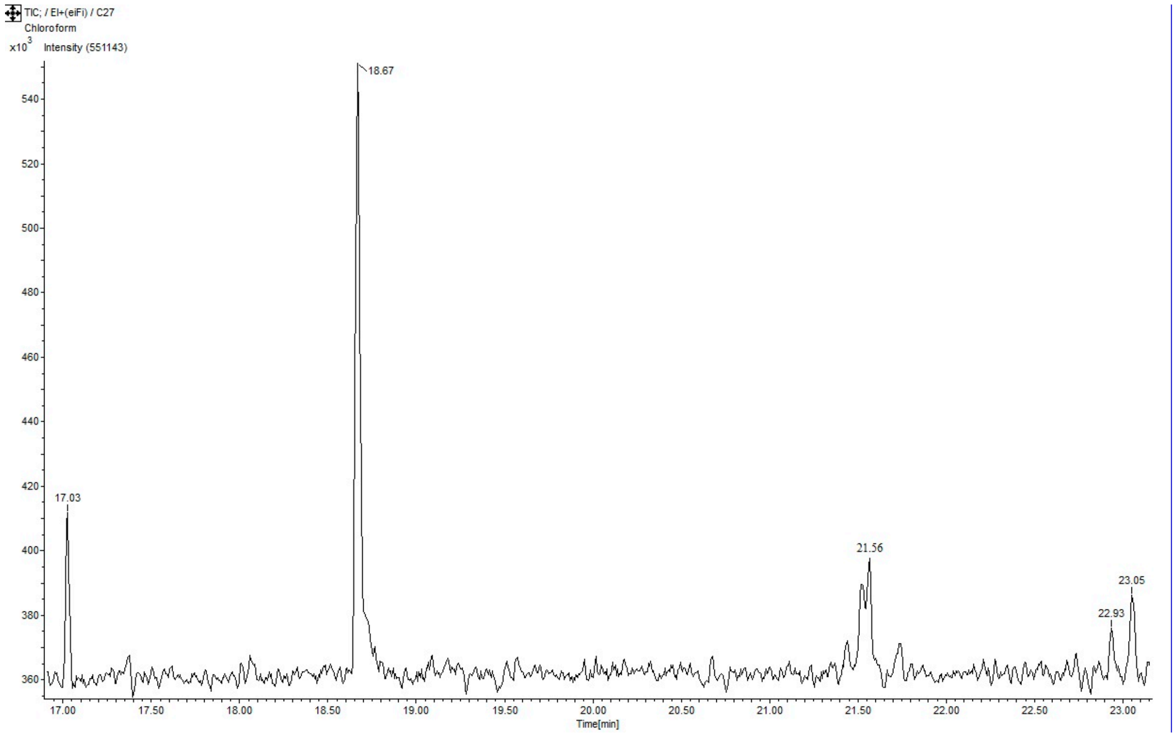

Figure S13. GC-MS spectrum of fraction F59–62.

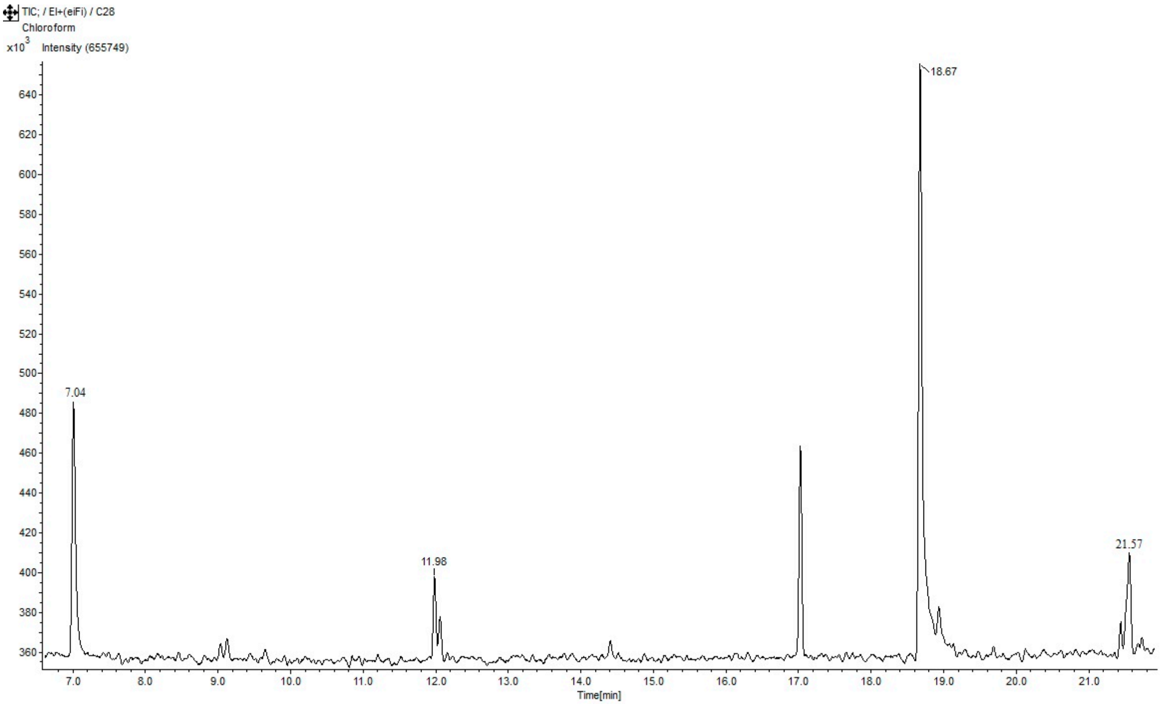

Figure S14. GC-MS spectrum of fraction F63–70.
